# Gut–brain axis in neurology: microbiome as a modifiable risk factor for multiple sclerosis

**DOI:** 10.1097/MS9.0000000000004648

**Published:** 2026-01-06

**Authors:** Mir R. Ali, Saniya Devi, Maria Jawed, Noor U.N. Irshad, Sakan B. Imran

**Affiliations:** a Department of Medicine, Jinnah Postgraduate Medical Centre, Karachi, Pakistan; b Department of Medicine, Jinnah Sindh Medical University, Karachi, Pakistan; c Department of Medicine, Liaquat University of Medical and Health Sciences, Jamshoro, Pakistan; d Department of Medicine, Sir Salimullah Medical College, Dhaka, Bangladesh

*Dear Editor,*

Our understanding of the gut–brain axis has grown over the past few years, as it is now known to have a quantifiable impact on the risk and progression of multiple sclerosis (MS) and that the gut microbiota may be able to be altered to influence the biology of the illness^[1,2]^. As clinicians, we believe that recognizing the microbiome as a practical and modifiable target is essential for future MS care. The International Multiple Sclerosis Microbiome Study and multiple meta-analyses have demonstrated changes in gut taxa in MS patients, including enrichment of *Akkermansia* and depletion of short-chain fatty acid (SCFA) producers such as *Faecalibacterium*, correlated with treatment and clinicaNot needed for LTEl outcomes^[2,3]^.

The mechanisms by which gut communities influence Central Nervous system (CNS) autoimmunity are plausible. Microbial metabolites derived from dietary substrates, particularly SCFAs and tryptophan metabolites, influence immune programming, regulatory T-cell induction, blood–brain barrier integrity, and CNS resident cell function^[1,4]^. Human observational studies connect microbial profiles with relapse risk and treatment response, whereas animal models confirm that fecal transplants from MS patients can exacerbate experimental autoimmune encephalomyelitis^[2,5]^.

Importantly, Mendelian randomization and multi-cohort analyses support a causal interpretation: genetic instruments link specific bacterial taxa, including *Ruminococcus torques*, to altered MS susceptibility, implying microbiome composition may contribute to disease risk^[5]^. To maintain clarity, only key reproducible dysbiosis patterns reduced *Faecalibacterium*, altered *Prevotella*/*Bacteroides* relationships, and increased oral-origin taxa are highlighted here^[3]^.

The microbiome remains appealing for translation because it can be altered by relatively low-risk methods such as diet, probiotics, prebiotics, defined microbial consortia, and fecal microbiota transplantation. Small clinical studies and mechanistic reviews demonstrate possible benefits of SCFA-promoting dietary interventions in restoring immune balance^[6,7]^. Nevertheless, inter-individual variability, host genetics, technical inconsistencies, and medication effects continue to limit predictable clinical responses^[1–3]^.

Therefore, practical next steps include integrating multi-omics datasets to identify responder phenotypes, conducting rigorously designed interventional trials, and accounting for dietary and medication confounders when testing microbiome-directed therapies^[2,3]^. From an author’s perspective, prioritizing microbiome-focused preventive strategies particularly in high-risk or early-stage individuals may become an important adjunct to current disease-modifying approaches. This article aligns with the TITAN Guidelines on transparency in AI use in health care^[8]^.

In conclusion, the gut microbiota represents a meaningful and modifiable factor in MS, supported by converging clinical, molecular, and causal-inference evidence. Opportunities for prevention and adjunct therapy arise from its influence on neuroinflammation, immune modulation, and barrier integrity. The microbiome is a practical therapeutic target given its responsiveness to diet and probiotics. We strongly argue that future research should prioritize identifying causal pathways and predictive microbial markers to support personalized, microbiome-integrated MS management.

## Data Availability

Not applicable.

## Research registration unique identifying number (UIN)

Not needed for LTE.
